# External validation of an integrative model of functional impairment in early-stage behavioral variant frontotemporal dementia (bvFTD)

**DOI:** 10.1007/s00415-026-14182-5

**Published:** 2026-10-05

**Authors:** Electra Chatzidimitriou, Despina Moraitou, Panagiotis Ioannidis, Eleni Aretouli, Yu Chen, Howard J. Rosen, Maria Luisa Gorno-Tempini, William W. Seeley, Bruce L. Miller, Katherine P. Rankin

**Affiliations:** 1 Department of Neurology, The Edward and Pearl Fein Memory and Aging Center, University of California, San Francisco, CA USA; 2 Department of Cognition, Brain and Behavior, School of Psychology, Faculty of Philosophy, Aristotle University of Thessaloniki, Thessaloniki, Greece; 3 2nd Department of Neurology, AHEPA University Hospital, Aristotle University of Thessaloniki, Thessaloniki, Greece; 4 Department of Psychology, School of Social Sciences, University of Ioannina, Ioannina, Greece

**Keywords:** BvFTD, Functional impairment, Path model, Cognitive deficits, Neuropsychiatric symptoms, Neuroimaging

## Abstract

**Background/Objectives:**

Functional decline in behavioral variant frontotemporal dementia (bvFTD) emerges early and significantly disrupts quality of life, yet its determinants remain incompletely understood. Integrative models capturing key disease manifestations may better explain its complexity but require validation in independent samples. This study aimed to examine whether a previously proposed model of functional impairment could be supported in a larger cohort and characterize the interrelationships among its cognitive, behavioral, personality, and neuroanatomical components.

**Methods:**

A total of 146 individuals with mild-stage bvFTD underwent structural MRI, neuropsychological assessment, and informant-based evaluation of behavioral symptoms, personality traits, and functional status. Penalized Least Absolute Shrinkage and Selection Operator (LASSO) regression identified the strongest correlates of functional impairment (Functional Activities Questionnaire/FAQ), while structural equation modeling examined their interrelationships.

**Results:**

LASSO identified conscientiousness, apathy, global cognition, executive functioning, sarcasm perception, and semantic verbal fluency as significant correlates of functional status. The resulting path model demonstrated excellent fit and supported both neural–cognitive and behavioral–personality pathways. Reduced right superior frontal gyrus volume was associated with poorer executive and global cognitive performance, and through these variables, with greater functional impairment. In parallel, apathy demonstrated both direct and indirect associations with functional decline through conscientiousness.

**Conclusions:**

Two partially distinct yet complementary processes appear to contribute to functional impairment in bvFTD: one reflecting the impact of frontal neurodegeneration on cognitive abilities essential for independent functioning, and another involving motivational and personality changes that directly undermine everyday engagement, thereby providing support for a multidimensional model of functional disability in bvFTD.

## Introduction

Behavioral variant frontotemporal dementia (bvFTD) represents the most prevalent clinical subtype of frontotemporal dementia (FTD) and a leading cause of early-onset dementia, typically affecting individuals younger than 65 years of age [1]. It constitutes a progressive neurodegenerative clinical syndrome characterized by early and prominent changes in personality, social conduct, motivation, and executive functioning [2]. In contrast to other dementia syndromes in which episodic memory deficits may dominate early clinical presentation, bvFTD frequently manifests through apathy, reduced empathy, disinhibition, compulsive behaviors, impaired judgment, and loss of insight [3]. These disturbances often translate rapidly into difficulties in activities of daily living (ADLs), including financial management, medication adherence, household organization, and independent community functioning [4–6]. As a result, functional impairment constitutes one of the most clinically meaningful outcomes in bvFTD, closely linked to caregiver burden, supervision needs, institutionalization risk, and reduced quality of life [6].

The determinants of functional decline in bvFTD appear to be complex and multidimensional [4]. Cognitive deficits, particularly in executive functioning, attentional control, episodic memory, and global cognition, are consistently associated with reduced everyday competence [5, 7–14]. However, cognition alone may not fully explain functional disability in bvFTD [6, 15]. Behavioral symptoms, such as apathy, may substantially diminish goal-directed behavior and initiative, even when basic cognitive capacities remain relatively preserved [5, 7, 9, 11–14, 16–18]. Similarly, alterations in personality traits, especially reduced conscientiousness, may impair organization, reliability, planning, and self-regulation in daily life [14]. Social cognitive deficits, including impaired emotion recognition, perspective taking, sarcasm detection, socioemotional semantic knowledge, and interpretation of interpersonal cues may further compromise real-world functioning by disrupting social judgment and adaptive decision-making [13, 19]. At the neurobiological level, frontal lobe degeneration––particularly involving prefrontal regions implicated in executive control, mental flexibility, attention, behavioral regulation, and motivation––may provide an anatomical substrate linking these clinical manifestations to everyday disability [4, 8, 14, 20, 21].

Despite growing recognition of these contributing factors, most previous research has often examined predictors of functional status in isolation. Studies have typically focused on either neuropsychological performance, neuropsychiatric symptoms, or structural neuroimaging markers, rather than evaluating their simultaneous and potentially overlapping contributions within a unified framework. This siloed approach may underestimate the multifactorial nature of disability in bvFTD. Multivariate integrative models are therefore particularly relevant [7], as they allow estimation of the independent associations of distinct domains while accounting for shared variance among correlated clinical features. Importantly, however, even when such models are developed, their generalizability remains uncertain unless they are tested in independent samples. External validation is therefore a critical but underutilized step in dementia research, as it determines whether observed relationships are robust beyond the original derivation cohort and transferable across clinical, demographic, and cultural contexts.

In response to the need for such unified frameworks, we previously conducted an exploratory study in a Greek cohort of 26 individuals with bvFTD, from which we developed a theoretical integrative model of functional decline incorporating cognitive, behavioral, personality-related, and neuroimaging variables [14]. Using multivariate analytic methods, that study identified several key correlates of functional impairment, including poorer global cognition, attentional and processing speed deficits, lower semantic verbal fluency, more severe negative behavioral symptoms such as apathy, and reduced conscientiousness. Importantly, the findings suggested that motivational and personality-related disturbances substantially contributed to functional limitations beyond the effects of cognitive performance alone, underscoring the importance of noncognitive dimensions in shaping everyday disability in bvFTD. The study also identified significant neuroimaging correlates of functional impairment, including reduced perfusion in right prefrontal regions, specifically Brodmann area (BA) 8, a region implicated in higher-order attentional regulation, behavioral control, motor planning, and the initiation and maintenance of goal-directed behavior—functions that are highly relevant to autonomy in everyday life [22].

Subsequently, a series of small path models were examined to explore the hypothesized direction of relationships among variables of interest and to characterize how these key correlates may interrelate and contribute to unique functional profiles in bvFTD. The confirmed path models suggested that lower right prefrontal perfusion was associated with poorer attentional performance and slower processing speed, and through these variables, with more severe negative behavioral symptoms. These behavioral manifestations were further associated with lower global cognition and reduced conscientiousness, with all domains ultimately relating to poorer daily functioning. A parallel pathway within the proposed model also linked right prefrontal abnormalities with reduced semantic verbal fluency, which was independently associated with functional limitations. Given the cross-sectional design of the study, these pathways were interpreted as patterns of association rather than causal relationships. Nevertheless, the findings provided a preliminary empirically informed integrative framework linking frontal neural dysfunction, cognitive deficits, behavioral disturbances, and personality changes to the functional phenotype of bvFTD.

Given the modest sample size of the original Greek cohort and the statistical challenges associated with simultaneously examining the complete hypothesized path model encompassing relationships among all the aforementioned variables, validation in a larger independent population was warranted. Accordingly, the present study aimed to examine whether the previously proposed preliminary framework could be supported in a substantially larger United States sample of individuals with early-stage bvFTD and to determine whether the original findings could be replicated and synthesized within a comprehensive path model.

Hence, the purpose of the present study was to examine whether previously identified associations between functional impairment and measures of cognition, behavior, socioemotional functioning, personality, and neuroanatomy, as well as their interconnections, would be reproduced in an independent cohort. Demonstrating cross-sample validity would support the robustness of a multidomain framework of functional impairment in bvFTD, enhancing understanding of the mechanisms underlying everyday disability and potentially informing refined strategies for both clinical assessment and intervention.

## Methods

### Participants

In this retrospective observational study, 146 individuals meeting the International bvFTD Criteria Consortium (FTDC) revised guidelines [2] for the diagnosis of at least possible bvFTD underwent comprehensive evaluation at the Edward and Pearl Fein Memory and Aging Center, Department of Neurology, University of California, San Francisco (UCSF). Diagnoses were established by a multidisciplinary team of neurologists, neuropsychologists, and nurses, following thorough neurological, neuroimaging, neuropsychological, genetic, and biomarker assessments.

To ensure inclusion of individuals in the early stages of disease and to exclude more advanced cases, only patients with a Mini-Mental State Examination (MMSE) score greater than 18 were included. This threshold was selected to maintain sufficient cognitive capacity for the reliable assessment of cognitive and socioemotional processes, while minimizing floor effects associated with more severe impairment. This criterion was further intended to focus the analyses on mild dementia, thereby reducing the confounding effects of advanced neurodegeneration, in which widespread deficits may obscure more specific brain–behavior relationships among variables of interest. Study participation additionally required the availability of a knowledgeable informant (e.g., close relative or friend), who knew the patient well and could provide reliable information regarding behavioral changes, personality alterations, and functional abilities.

Exclusion criteria included the presence of significant cerebrovascular lesions on magnetic resonance imaging (MRI) or computed tomography (CT), which could confound neuroimaging interpretation, as well as other neurological disorders capable of accounting for cognitive, behavioral, or functional impairment, including stroke, Parkinson’s disease, multiple sclerosis, epilepsy, or traumatic brain injury. Patients with major psychiatric disorders (e.g., schizophrenia, bipolar disorder, or major depressive disorder) were also excluded. Finally, individuals with severe sensory, motor, or physical impairments that could substantially interfere with the assessment of everyday functioning were not eligible for inclusion.

### Procedures

All participants underwent MRI and a comprehensive neuropsychological assessment at their earliest visits to the memory clinic. The neuropsychological evaluation comprised participant-administered face-to-face cognitive tests and informant-based measures assessing behavioral symptoms, personality traits, and functional status. All evaluations were conducted by certified clinical neuropsychologists with extensive experience in dementia research.

### Protocol approvals and patient consents

The study was approved by the UCSF Institutional Review Board for human research (#14–14044). All participants and their informants gave their written informed consent to participate and share data. All study procedures were conducted in accordance with the ethical standards of the 1964 Declaration of Helsinki and its later amendments.

### Neuropsychological and neuroimaging assessment

A key aspect of the study design was the deliberate selection of variables corresponding to those administered in the original Greek sample. Specifically, we aimed to operationalize comparable constructs across neuroimaging, cognitive, behavioral, socioemotional, and personality domains to evaluate the external validity of the previously proposed multivariate framework. Given that data were derived from independent samples across different centers, identical instruments were not always available. Therefore, variables were selected based on conceptual and psychometric equivalence rather than exact measurement overlap. In other words, we prioritized measures that indexed the same underlying constructs as those used in the original study to ensure conceptual alignment, even when different assessment tools were employed across sites. In some domains, identical instruments were available across cohorts (e.g., the Awareness of Social Inference Test—TASIT), whereas in other cases closely matched proxies were selected based on established construct validity and clinical comparability. This approach reflects the primary aim of the study, namely the external validation of a previously derived integrative model, rather than exploratory identification of novel correlates.

### Disease severity measures

Disease severity was assessed using both the Clinical Dementia Rating (CDR) scale [23] and its frontotemporal dementia–specific extension, the FTLD-specific CDR [24]. The CDR evaluates six domains (memory, orientation, judgment/problem solving, community affairs, home/hobbies, and personal care), yielding both a global score (CDR-GS; 0–3) and a sum of boxes score (CDR-SB; 0–18), with higher scores indicating greater impairment. The FTLD-specific CDR extends this framework by including additional domains assessing language and behavior, thereby improving sensitivity to frontotemporal syndromes. It similarly provides a global score (FTLD-CDR-GS; 0–3) and a composite sum of boxes score (FTLD-CDR-SB; 0–24), with higher values reflecting greater disease severity.

### Cognitive assessment

Global cognitive functioning was assessed using the MMSE [25], a well-known and widely used screening instrument comprising tasks evaluating orientation, attention, memory, language, and visuoconstructional abilities. Total scores range from 0 to 30, with higher scores indicating better cognitive performance.

Executive functioning was examined through measures of cognitive flexibility and inhibitory control. Cognitive flexibility was assessed using a modified version of the Trail Making Test (TMT), requiring participants to alternate sequentially between numbers and days of the week under timed conditions (maximum duration: 120 s) [26, 27]. Performance was indexed by completion time, with longer times reflecting poorer executive efficiency. Response inhibition was assessed using the interference condition of the Stroop Test, with performance defined as the number of correct responses within 60 s [27, 28].

Semantic retrieval and verbal productivity were assessed using a semantic verbal fluency task. Participants were instructed to generate as many words as possible belonging to a given semantic category (animals) within 60 s [27]. The total number of correct responses constituted the performance score, with higher scores indicating better semantic verbal fluency.

Visuoconstructional abilities were evaluated using the Benson Complex Figure task (copy condition) [27, 29], in which participants were asked to reproduce a complex geometric figure. Performance was scored based on the accuracy and placement of figure components (range: 0–17), with higher scores indicating better visuoconstructional performance. Additional visuospatial ability was assessed using a pentagon-copying task (scored 0–1).

As regards social cognition, sarcasm perception was assessed using the Social Inference–Minimal subtest of “The Awareness of Social Inference Test” (TASIT) (TASIT-SI-M) [30]. This measure examines the ability to differentiate sincere from sarcastic exchanges and to infer the speaker’s intended meaning based on visual and vocal paralinguistic cues, including facial expressions, voice prosody, body posture, and hand or head gestures. Participants viewed 15 brief video vignettes depicting naturalistic social interactions involving sincere, simple sarcastic, or paradoxical sarcastic exchanges (five items per condition). After each vignette, participants answered four yes-or-no questions concerning the characters’ actions, thoughts, words, and emotions. For the purposes of the present study, only the simple sarcasm summary score was analyzed (maximum score = 20), with higher scores reflecting better performance in detecting the paralinguistic signals of sarcasm without additional contextual or content cues.

### Personality assessment

Current personality traits were assessed using the informant-rated version of the Big Five Inventory (BFI) [31], a 44-item questionnaire measuring five major personality dimensions: Agreeableness, Conscientiousness, Extraversion, Neuroticism, and Openness to Experience. Agreeableness reflects interpersonal trust, altruism, cooperativeness, compliance, empathy, and concern for others; Conscientiousness assesses organization, self-discipline, responsibility, achievement striving, and goal-directed behavior; Extraversion captures sociability, gregariousness, assertiveness, activity, and positive emotionality; Neuroticism reflects tendencies toward anxiety, emotional instability, and vulnerability; and Openness to Experience encompasses intellectual curiosity, imagination, esthetic sensitivity, and receptiveness to new experiences. Items are rated on a 5-point Likert scale ranging from 1 (“disagree strongly”) to 5 (“agree strongly”), with higher scores indicating greater endorsement of the corresponding personality characteristic. Given the reduced insight and self-awareness frequently observed in bvFTD [2, 32–34], informant-based ratings were used, as they are generally considered more reliable than self-reports in this population. Responses were summed to generate composite scores for each personality domain, with higher scores indicating greater expression of the corresponding trait and lower scores reflecting reduced expression. Composite scores for all five personality dimensions were included in the analyses of the present study.

### Neuropsychiatric assessment

At the level of behavioral and neuropsychiatric assessment, the Neuropsychiatric Inventory (NPI) [35] was administered to evaluate the presence and severity of behavioral symptoms commonly observed in neurodegenerative disorders. The NPI assesses 12 domains, including delusions, hallucinations, agitation/aggression, depression/dysphoria, anxiety, elation/euphoria, apathy/indifference, disinhibition, irritability/lability, aberrant motor behavior, sleep/night-time behavior disorders, and appetite/eating changes. Each domain is rated based on frequency (1–4) and severity (1–3), yielding composite scores ranging from 0 to 12, with higher scores reflecting more pronounced behavioral disturbances. For the purposes of the present study, the apathy/indifference and disinhibition domain scores were included in the analyses.

### Functional measures

The Functional Activities Questionnaire (FAQ) [36] was used as the primary outcome measure to assess functional status and quantify impairments in instrumental activities of daily living (IADLs). The FAQ is an informant-based questionnaire comprising 10 items, each rated on a 4-point Likert scale reflecting levels of independence: 0 = normal, 1 = has difficulty but performs independently, 2 = requires assistance, and 3 = completely dependent. Informants were asked to evaluate participants’ functioning over the preceding four weeks across ten domains of daily activities, including financial management (e.g., writing checks, paying bills, balancing a checkbook), handling tax or business affairs, independent shopping, engagement in hobbies or games of skill, basic household tasks (e.g., preparing meals, operating kitchen appliances), keeping track of current events, comprehension and discussion of television programs, books or magazines, memory for appointments and important occasions, and independent transportation. Item scores were summed to yield a total FAQ score ranging from 0 to 30, with higher scores indicating greater functional impairment and reduced independence in everyday activities.

### Structural MRI acquisition and preprocessing steps

MRI scans were acquired within 90 days of the neuropsychological and functional assessment using 3 Tesla Siemens scanners (Siemens Trio and Prisma) at the UCSF Neuroscience Imaging Center. T1-weighted three-dimensional magnetization-prepared rapid gradient echo (MPRAGE) sequences were obtained with the following parameters: 160 sagittal slices (1 mm isotropic resolution, no gaps), repetition time = 2300 ms, echo time = 2.98 ms, flip angle = 9°, field of view = 240 × 256 mm^2^, and matrix size = 256 × 256. Structural T1-weighted image preprocessing was performed using Statistical Parametric Mapping software (SPM12) [37]. Images underwent visual inspection for artifacts, followed by bias-field correction, tissue segmentation, and spatial normalization using a unified generative model implemented in SPM12 with default parameters. Default tissue probability maps for gray matter, white matter, cerebrospinal fluid, and all other voxels from SPM12 (TPM.nii) were utilized [37]. The total volume for each tissue compartment was derived by applying the modulated, warped, and segmented (MWS) masks for gray matter, white matter, and cerebrospinal fluid to the respective MWS probability map for each individual, with the total intracranial volume (TIV) calculated as the sum of these three volumes. The Neuromorphometrics atlas was then applied to the spatially normalized, segmented, and modulated gray matter images, allowing for the subsequent extraction of region of interest (ROI) volumes using voxel-based morphometry (VBM) [19, 37, 38].

For the purposes of the present study, analyses focused on the gray matter volume of the right superior frontal gyrus (SFG), selected based on its correspondence with findings from the original Greek cohort [14]. In the original Greek cohort, right BA 8 emerged as the strongest neural correlate of functional status and therefore served as the neuroimaging reference region for the present external validation study. Because comparable perfusion imaging data with BA-level analysis were not available in the present independent cohort, a structurally corresponding region was identified using the Neuromorphometrics atlas. Specifically, based on the known anatomical localization of right BA 8 in the existing neuroanatomical literature, the right SFG was selected as the closest anatomically corresponding structural region available in the Neuromorphometrics atlas. Thus, the right SFG was defined a priori as the anatomically based ROI for the present study, representing an anatomically informed regional approximation, rather than being derived from a voxel-wise VBM analysis performed in this cohort. The mean voxel intensity within the right SFG ROI was extracted for each participant to obtain the regional gray matter volume, which was then normalized for TIV by dividing it by the individual’s TIV, thereby accounting for inter-individual differences in overall intracranial size. The resulting TIV-normalized SFG volumes were used in all subsequent statistical analyses.

The SFG, located at the superior part of the prefrontal cortex, is implicated in multiple higher-order functions, including executive control, attentional regulation, motor planning, and the initiation and monitoring of goal-directed behavior. Its involvement in cognitive, emotional, and behavioral regulation renders it a key candidate region within the neural architecture underlying functional impairment in bvFTD [20, 39, 40].

### Statistical analyses

All data preprocessing, statistical analyses, and visualization were conducted using the R programming language and statistical computing environment (R Foundation for Statistical Computing, Vienna, Austria; version 4.5.1; released in June 2025) [41].

Missing data in cognitive and behavioral variables were handled using multiple imputation via the “Multivariate Imputation by Chained Equations” (MICE) method, implemented through the *“mice”* package in R [42, 43]. MICE is a well-established and widely used approach in the biostatistical literature that enables the estimation of missing values based on observed relationships among variables, thereby reducing potential bias and preserving statistical power under the assumption that data are missing at random (MAR). A total of 50 imputed datasets were generated using predictive mean matching (PMM) with 20 iterations. Multiple imputation was applied exclusively to predictor variables to ensure complete data availability for subsequent regression and path modeling analyses. Imputation was not applied to the primary outcome measure, the FAQ scale, as complete functional data were available for all participants.

Descriptive statistics were computed to summarize participants’ baseline demographic and clinical characteristics. Additionally, data were examined for outliers using standardized z-scores and visual inspection of boxplots, with extreme values assessed for potential exclusion. A *p*-value < 0.05 was initially considered the threshold for statistical significance. To account for multiple comparisons, the Bonferroni correction was applied by dividing the significance level (α = 0.05) by the number of variables assessed (16 variables), resulting in an adjusted significance level of 0.0031. In all analyses, statistical significance was then determined using this adjusted threshold to control the family-wise error rate (FWER) and reduce the risk of Type I errors.

To identify the most robust correlates of functional impairment in individuals with bvFTD, we first employed penalized Least Absolute Shrinkage and Selection Operator (LASSO) regression analysis to perform data-driven variable selection. This approach allowed us to examine the most relevant correlates of functional status while accounting for potential multicollinearity among candidate variables. Subsequently, path analysis was conducted to investigate the interrelationships among variables of interest and to model their direct and indirect associations with functional impairment.

LASSO regression was implemented in R using the *“glmnet”* package, with the alpha parameter set to 1 to specify L1 regularization [41]. LASSO is widely used in predictive modeling and machine learning due to its ability to handle high-dimensional data by shrinking noninformative coefficients toward zero, thereby facilitating variable selection and improving model interpretability [44–46]. The dataset was randomly partitioned into training (70%) and test (30%) subsets for model development and performance evaluation. The optimal regularization parameter (λ) was determined through a 10-fold cross-validation using the *“cv.glmnet”* function, based on minimization of mean squared error (MSE). The final model was then refitted using the optimal λ, and regression coefficients were extracted and visualized to identify the most informative correlates of functional performance.

Subsequently, path analysis was conducted using EQS version 6.4 [47] to examine the structural relationships among variables of interest and functional status. Structural equation modeling (SEM) based on covariance matrices was employed using maximum likelihood (ML) estimation. In the SEM analysis, all candidate variables were initially considered for inclusion. Thus, LASSO selection was not used as an inclusion criterion for the path model. This analytic approach was considered appropriate because some candidate variables may not demonstrate an independent direct contribution to FAQ scores in the LASSO analysis but may nevertheless have an important role in the path model through their relationships with other variables, contributing indirectly to functional status.

Model fit was considered acceptable based on a combination of indices: a nonsignificant chi-square test (χ^2^, *p* > 0.05) indicating adequate model fit; the Root Mean Square Error of Approximation (RMSEA), with values ≤0.05 indicating good fit and values between 0.06 and 0.08 indicating acceptable fit; the Comparative Fit Index (CFI), with values ≥0.95 indicating good fit and ≥0.90 acceptable fit; and the Standardized Root Mean Square Residual (SRMR), with values <0.05 indicating good fit and <0.08 acceptable fit. Different path model configurations were evaluated and compared based on their fit to the observed data to identify the model that provided the best fit based on these predefined fit indices. Through this model-fitting process, variables that did not contribute meaningfully to the broader structural relationships were not retained in the final model.

## Results

### Demographic and clinical characteristics

The sample consisted of 146 individuals diagnosed with bvFTD, with a distribution of 97 males (66.40%) and 49 females (33.60%). The mean age at assessment was 61.77 years (SD = 9.13), with a mean age at symptom onset of 54.87 years (SD = 8.07). In terms of educational attainment, participants had a mean of 16.05 years of education (SD = 2.75).

Clinical assessment indicated a mean CDR-GS of 1.01 (SD = 0.52) and a mean CDR-SB score of 5.62 (SD = 2.75), consistent with mild dementia severity. As expected, impairment was more pronounced when assessed using the FTLD-specific scale, with a mean FTLD-CDR-GS of 1.48 (SD = 0.65) and a mean FTLD-CDR-SB score of 7.65 (SD = 3.33), reflecting greater sensitivity to frontotemporal dysfunction. Cognitive performance, as measured by the MMSE, showed a mean score of 25.45 (SD = 3.41), indicating mild cognitive impairment. Table 1 summarizes the demographic and clinical characteristics of the study sample.

**Table 1:** Demographic and clinical characteristics of participants

Mean (SD)	bvFTD patients
n	146
Age, years	61.77 (9.13)
Age at symptom onset, years	54.87 (8.07)
Sex, M/F, n	97/49
Education, years	16.05 (2.75)
MMSE total score	25.45 (3.41)
CDR sum of boxes	5.62 (2.75)
CDR global score	1.01 (0.52)
FTLD-CDR sum of boxes	7.65 (3.33)
FTLD-CDR global score	1.48 (0.65)

### Results of the penalized LASSO regression analysis

Penalized LASSO regression analysis was performed to identify the strongest correlates of functional impairment, with the FAQ score entered as the dependent variable (outcome measure) and cognitive, socioemotional, behavioral, personality, and neuroanatomical variables entered as candidate predictors.

The optimal regularization parameter (λ) identified through the 10-fold cross-validation procedure was 0.403, which minimized the cross-validated MSE.

**Fig. 1:**
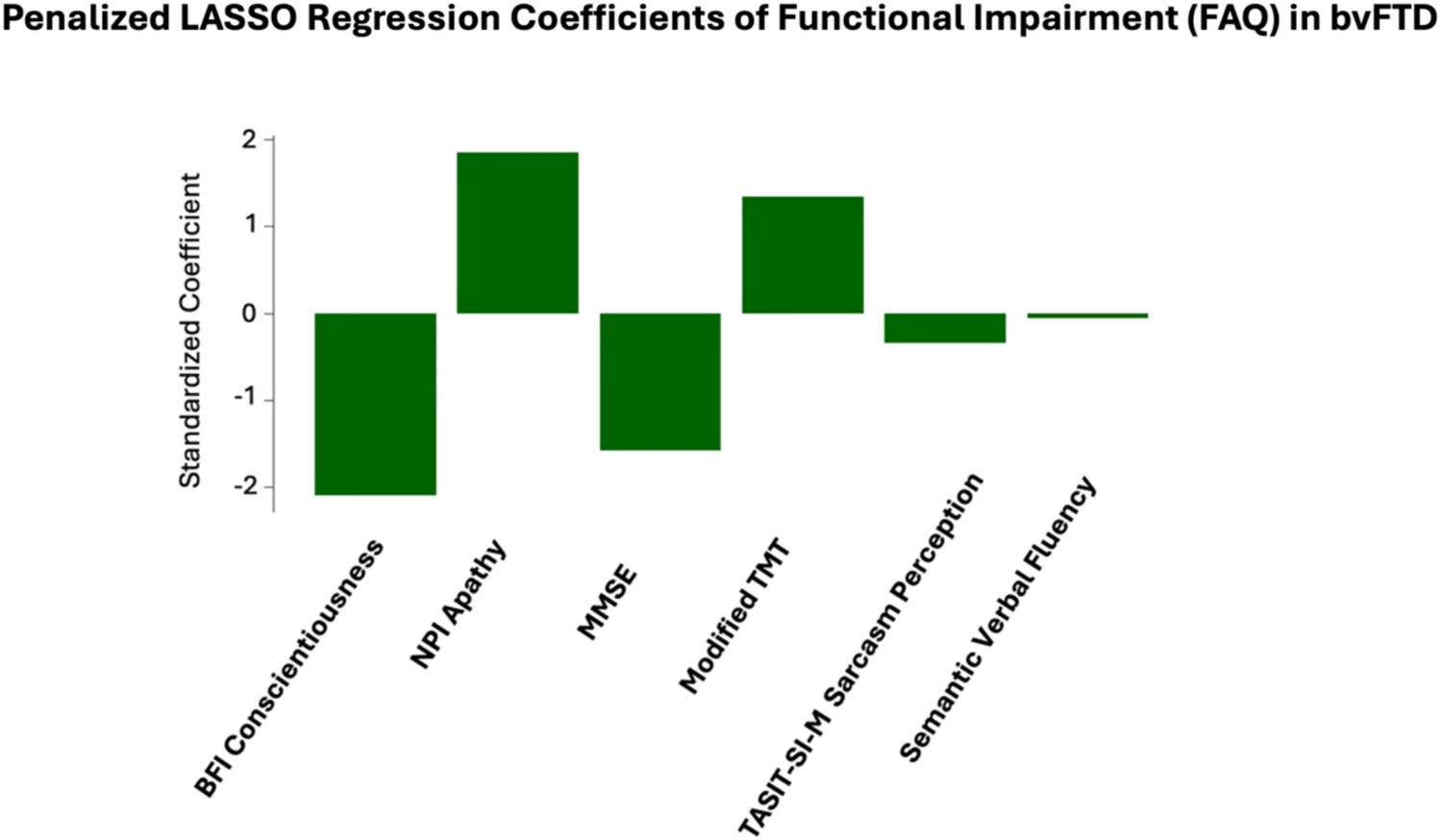
Bar chart illustrating the coefficient estimates of the predictors included in the final penalized LASSO regression model for the FAQ total score. Predictors are presented in descending order based on the absolute values of their relative contribution to the model. Abbreviations (in alphabetical order): *BFI* Big Five Inventory, *MMSE* Mini-Mental State Examination, *NPI* Neuropsychiatric Inventory, *TASIT-SI-M* the Awareness of Social Inference Test—Minimal Subtest, *TMT* Trail Making Test

The final LASSO regression model identified BFI Conscientiousness (β =  − 2.10), NPI Apathy (β = 1.84), MMSE (β =  − 1.58), Modified TMT (β = 1.34), TASIT – Sarcasm Perception (β =  − 0.34), and Semantic Verbal Fluency (β =  − 0.06) as significant correlates of FAQ scores in bvFTD (*p* < 0.001) (see Fig. 1).

The remaining candidate predictors, including the Benson Complex Figure Test – Copy Condition, Pentagons, Stroop Test, NPI Disinhibition, BFI Agreeableness, BFI Extraversion, BFI Neuroticism, BFI Openness to Experience, and right SFG volume, were excluded from the final model, as their coefficients were shrunk to zero, indicating no independent contribution to the prediction of FAQ scores.

The final LASSO regression model yielded an MSE of 34.92, indicating the average squared difference between the observed and predicted values. Additionally, the model explained approximately 39.3% of the variance in FAQ scores, with an *R*^2^ value of 0.393.

### Path analysis results

Based on existing literature regarding the relationships among the variables of interest, as well as the theoretical framework proposed in our previous study, the final path model supported in the independent validation cohort is presented below.

**Fig. 2:**
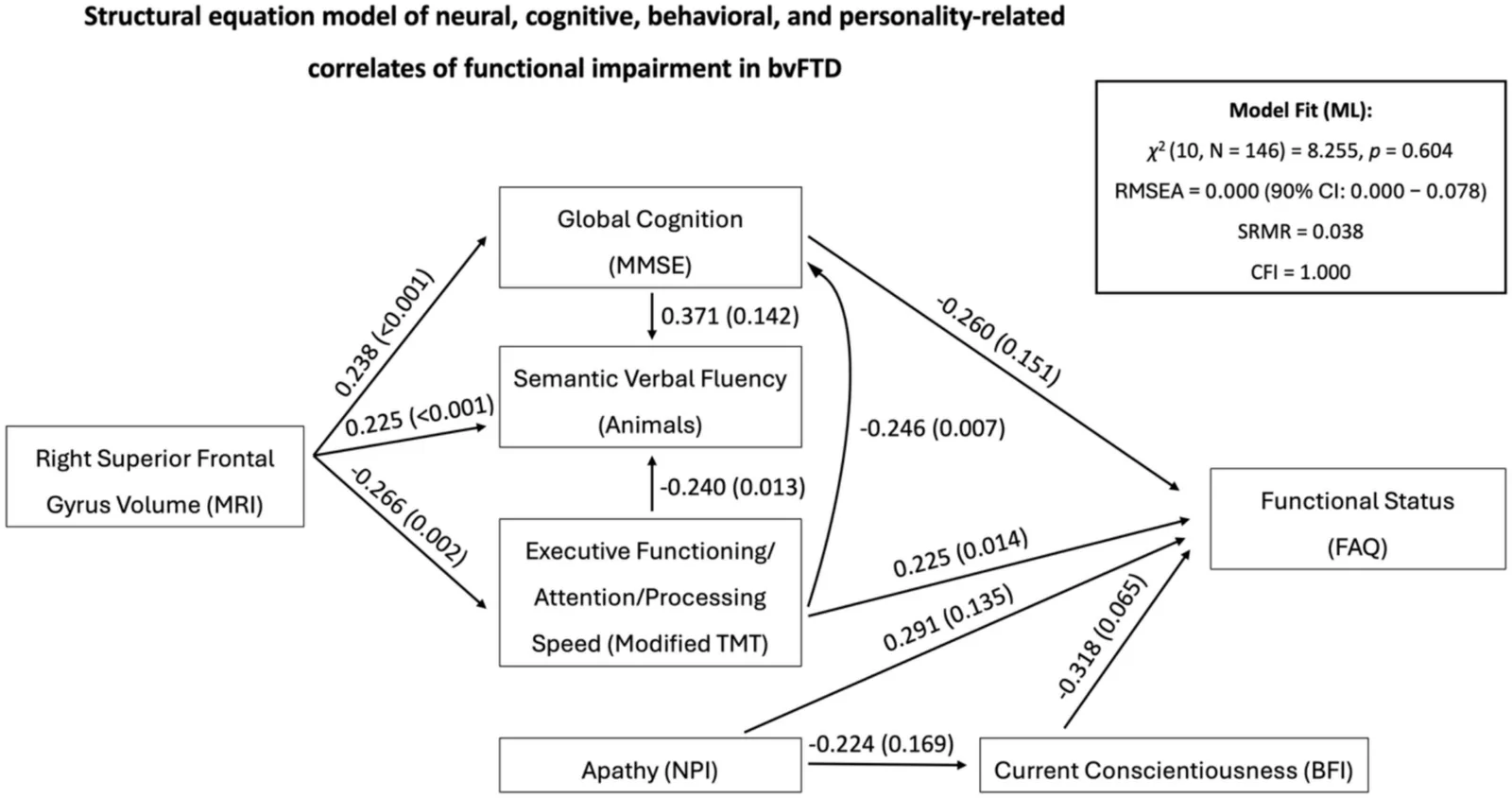
Confirmed path analysis model illustrating the relationships among neural, cognitive, behavioral, and personality-related correlates of functional status in bvFTD. Standardized path coefficients (β) are displayed above the arrows, with measurement errors indicated in parentheses. Abbreviations (alphabetical order): *BFI* Big Five Inventory, *bvFTD* behavioral variant frontotemporal dementia, *FAQ* Functional Activities Questionnaire, *MMSE* Mini-Mental State Examination, *MRI* magnetic resonance imaging, *NPI* Neuropsychiatric Inventory, *TMT* Trail Making Test

The final SEM model, depicting the direction of interrelationships among the variables of interest, demonstrated excellent overall fit to the observed data: *χ*^2^(10, *N* = 146) = 8.255, *p* = 0.604, CFI = 1.000, RMSEA = 0.000 (90% CI 0.000–0.078), and SRMR = 0.038 (see Fig. 2).

Although 15 variables were initially considered as potential predictors, the final parsimonious model retained six predictors, including right SFG volume, modified TMT completion time, MMSE, semantic verbal fluency, BFI conscientiousness, and NPI apathy.

### Structural pathways

As shown in Fig. 2, three brain–cognition pathways were identified as contributing to functional status. First, a statistically significant path was identified from right SFG volume through Modified TMT completion time to FAQ, indicating an indirect pathway whereby reduced right SFG volume was associated with lower executive functioning and poorer functional status. Second, a path from right SFG volume through MMSE to FAQ was identified, suggesting a pathway whereby lower right SFG volume was directly associated with lower global cognition, and through this variable, with worse functional status. A third path was also revealed, including the effects of right SFG volume via Modified TMT completion time to MMSE and through these two cognitive measures to FAQ, indicating that the indirect relationships between right SFG volume and FAQ scores are additionally characterized by the effects of executive functioning on global cognitive performance.

With respect to semantic verbal fluency, direct paths were identified from right SFG volume, Modified TMT completion time, and MMSE. However, semantic verbal fluency was incorporated within the model as an endogenous cognitive variable and did not demonstrate a direct path to FAQ in the final retained structure.

Regarding behavioral–personality pathways contributing to functional status, a path from NPI Apathy through BFI Conscientiousness to FAQ was revealed, indicating that higher levels of apathy were related to greater functional impairment through their association with lower conscientiousness. However, NPI Apathy also demonstrated a direct link with FAQ, suggesting that apathy contributed to functional impairment both directly and indirectly.

The model accounted for 38.2% of the variance in FAQ (*R*^2^ = 0.382). Variance explained for intermediate endogenous variables was 38.0% for semantic verbal fluency, 14.9% for MMSE, 7.1% for Modified TMT completion time, and 5.0% for BFI conscientiousness.

## Discussion

The present study aimed to examine whether a previously proposed integrative model of functional impairment in bvFTD could be supported in a larger independent sample, by evaluating the relative contributions and interrelationships among cognitive, behavioral, personality-related, and neural variables. Overall, the findings are broadly consistent with those reported in the original Greek sample [14] and reinforce a multidomain framework of functional disability in bvFTD, highlighting the combined contribution of neural, executive, motivational, and personality-related factors.

### Multidomain correlates of functional status: evidence from LASSO

Prior to examining the structural pathways underlying functional decline, penalized LASSO regression analysis was used to identify the most robust independent correlates of functional impairment, with the final model retaining BFI Conscientiousness, NPI Apathy, MMSE, Modified TMT completion time, TASIT-SI-M sarcasm perception, and semantic verbal fluency as key variables. Specifically, higher levels of apathy and slower executive processing speed were associated with greater functional impairment, whereas higher conscientiousness, better global cognition, and stronger performance in social cognition (sarcasm perception) and semantic verbal fluency were associated with better functional status. Together, these variables accounted for a substantial proportion of variance in functional status, further supporting the multidimensional nature of everyday disability in bvFTD.

Notably, behavioral and personality-related variables—particularly apathy and conscientiousness—emerged as the strongest contributors in the model, followed by cognitive factors. This finding is highly consistent with the clinical phenotype of bvFTD, in which motivational and personality disturbances predominate, representing core and early features of the disease [2]. Additionally, it aligns with previous work emphasizing the importance of these factors in shaping everyday functioning, often outweighing cognitive impairment [5, 7, 9, 11–14, 16–18, 48]. Apathy, characterized by reduced engagement in previously meaningful activities, diminished goal-directed behavior, emotional blunting, and decreased responsiveness to environmental demands [49], directly undermines engagement in everyday activities. Similarly, reduced conscientiousness—reflecting impairments in organization, persistence, and self-regulation—can compromise the ability to plan, structure, and complete daily activities [14, 50].

Cognitive variables, including MMSE and Modified TMT performance, also contributed substantially, underscoring the importance of global cognition [9, 10, 12, 14, 48] and executive functioning [5, 8, 11, 13, 14, 48] in maintaining functional independence. Deficits in cognitive flexibility, set-shifting, attentional control, and processing speed can significantly disrupt the sequencing, monitoring, and adaptation required for complex everyday tasks [4, 51].

Importantly, sarcasm perception also emerged as a significant correlate of functional status, highlighting the relevance of social-cognitive and socioemotional processing deficits to functional impairment in bvFTD [13, 19]. The ability to effectively perceive and interpret paralinguistic and contextual cues is critical for effective interpersonal functioning, and its disruption may lead to misunderstandings, inappropriate responses, and impaired social decision-making [13, 19, 52–55].

Finally, semantic verbal fluency was also identified as an important cognitive correlate. This finding highlights the contribution of preserved semantic knowledge to functional abilities [14, 19, 56, 57], as well as the role of higher-order executive processes, including the efficient retrieval, organization, selection, and use of conceptual knowledge. Semantic systems provide the conceptual scaffolding necessary for interpreting environmental cues (e.g., understanding task demands) and generating contextually appropriate responses, capacities that are necessary for maintaining autonomy in daily living [14, 19, 56, 57].

In contrast, visuoconstructional skills, visuospatial performance, inhibitory control, and right SFG volume were not included in the final LASSO model. This may reflect shared variance with other predictors or a more indirect contribution to functional impairment. For example, aspects of inhibitory control may overlap with broader executive functions captured by Modified TMT performance or may be less sensitive to variability in functional outcomes when considered alongside measures of cognitive flexibility. Similarly, visuospatial and visuoconstructional abilities may be relatively less central to the core cognitive phenotype of bvFTD, particularly in the early stages of the disease, where they may be relatively preserved [2]. Structural neuroimaging measures may also influence functional outcomes indirectly, exerting their effects through downstream cognitive and behavioral processes rather than acting as direct predictors of functional status.

### Validation of the structural model: an integrative multivariate framework

Path analysis extended these findings by examining how neural, cognitive, behavioral, and personality-related variables are interconnected within a unified framework. The confirmed SEM demonstrated excellent fit to the data, supporting the adequacy and robustness of the proposed integrative model.

Consistent with the hypothesized framework as well as prior findings from the Greek sample [14], the model was anchored in prefrontal cortical structures, particularly the SFG, as an upstream neural factor. This region, implicated in cognitive, behavioral, and emotional regulation, as well as motor planning, plays a central role in attentional processes and executive control [22, 39, 40, 58].

Specifically, three pathways originating from right SFG volume were identified. First, reduced right prefrontal volume was associated with slower executive performance measured by modified TMT, which in turn predicted worse functional status. This pathway emphasizes the critical role of attentional control, cognitive flexibility, and executive speed in everyday functioning [5, 8, 11, 13, 14, 48]. Deficits in these processes may impair the ability to sustain cognitive effort, shift between tasks, adapt to changing demands, and coordinate multistep activities. For example, an individual may struggle to alternate between cooking tasks, monitor timing, or adjust actions in response to unexpected situations.

Second, a parallel pathway linked right SFG volume to functional disability through MMSE. This finding highlights the contribution of global cognitive functioning to everyday abilities, as reductions in overall cognitive capacity may compromise the integration of multiple processes required for independent functioning. Impairments in global cognition can affect memory, attention, orientation, and judgment, thereby reducing the ability to manage complex daily tasks, maintain routines, and respond appropriately to environmental demands [9, 10, 12, 14, 48]. Interestingly, the observed path from Modified TMT to MMSE suggests a hierarchical relationship in which executive dysfunction contributes to broader global cognitive decline. This aligns with the characteristic trajectory of bvFTD, in which early executive deficits, resulting primarily from frontal network disruption [2, 3, 21], progressively impact multiple cognitive domains, leading to more generalized impairment.

Another important finding concerns semantic verbal fluency. In the present model, semantic fluency was significantly associated with right SFG volume, Modified TMT completion time, and MMSE performance, although it did not demonstrate a direct path to functional status. This pattern can be considered within the framework of controlled semantic cognition (CSC) [59, 60], which proposes that semantic processing depends on the interaction between a semantic representational system supporting conceptual knowledge and a control system regulating the retrieval and selection of semantic information in a goal-directed and context-sensitive manner. Within this framework, MMSE may capture aspects of broader cognitive integrity, particularly memory-related components of the representational system, whereas Modified TMT may reflect executive control processes. The association of semantic verbal fluency with both measures is consistent with the complex nature of this cognitive task, which requires both access to semantic knowledge and executive processes involved in strategic retrieval, monitoring, and response generation. The absence of a direct association with functional status in the present multivariate model may suggest that semantic fluency operates as an intermediate cognitive marker, reflecting upstream disruptions in executive and global cognitive systems, which themselves more directly relate to functional outcomes. This finding also provides a potential explanation for discrepancies with our previous Greek study [14], in which semantic verbal fluency demonstrated a direct association with functional status. It is possible that, in smaller samples, semantic fluency captured shared variance across executive and global cognitive domains, whereas in the present larger cohort these relationships were more precisely disentangled.

The proposed model further identified a distinct behavioral–personality pathway linking NPI Apathy, BFI Conscientiousness, and FAQ. Apathy demonstrated both a direct association with functional impairment and an indirect link through conscientiousness, suggesting that motivational disturbances in bvFTD may relate to functional abilities both independently and via their impact on personality-related processes.

Apathy is one of the most prevalent and characteristic behavioral symptoms of bvFTD [2], with a particularly pronounced impact on autonomy and everyday functioning [5, 7–9, 11–14, 16–18, 48]. Individuals with bvFTD may neglect personal hygiene, meal preparation and household responsibilities, withdraw from social interactions and occupational roles, or show poor adherence to medication regimens, all of which can directly contribute to loss of functional independence. Conscientiousness, in turn, reflects the capacity for organization, persistence, goal-directed behavior, achievement orientation, and self-monitoring [31, 50]. Reductions in conscientiousness [61, 62] may be expressed as diminished sense of responsibility, reduced reliability, and impaired self-generated goal setting and maintenance, with consequent difficulties in maintaining routines and completing tasks effectively [14]. For instance, individuals may struggle to manage finances or pay bills on time, fail to keep scheduled appointments, demonstrate poor organization in household tasks, or be unable to plan and complete multistep activities such as shopping, cooking, or managing personal affairs. Ιn occupational contexts, where consistency, organization, and goal commitment are essential, reduced conscientiousness may manifest as decreased productivity, inability to meet deadlines, and loss of consistency and accountability, ultimately compromising work performance and social credibility. Within this framework, the observed pathway from apathy to conscientiousness suggests that motivational disturbances may disrupt the processes that support sustained, organized, and goal-directed behavior. Individuals with higher levels of apathy may be less likely to set goals, initiate actions, or persist in their efforts, which in turn may be reflected as lower conscientiousness. For example, a patient who does not initiate activities or maintain effort toward task completion may appear disorganized, inconsistent, and less dependable, ultimately compromising the effective planning and execution of everyday activities.

Interestingly, in the proposed model, this pathway appeared relatively independent of the neural–cognitive pathways, potentially indicating that behavioral–motivational and personality changes in bvFTD are mediated by partially distinct neural systems, such as medial frontal and limbic circuits [21, 63]. This finding is also consistent with previous reports supporting a dissociation between apathy and cognitive deficits, as well as with conceptualizations of apathy as a primary neuropsychiatric syndrome characterized by motivational loss, not attributable to intellectual impairment or emotional distress [49]. In this context, apathy in bvFTD appears to reflect a fundamental disruption in the initiation and maintenance of behavior rather than a downstream effect of executive dysfunction. This interpretation reinforces the notion that negative behavioral symptoms in bvFTD are not merely epiphenomena of cognitive deficits, but instead, constitute core drivers of functional decline.

### Clinical implications

These findings have important implications for clinical practice. First, the prominence of behavioral and personality predictors highlights the need for holistic assessments that extend beyond cognitive testing. In this context, clinical evaluation should move beyond isolated cognitive screening and adopt a more comprehensive approach that integrates neurocognitive, behavioral, socioemotional, and personality domains, as functional impairment in bvFTD cannot be adequately captured through cognitive assessment alone [6, 7, 14, 15, 48].

Care planning and intervention strategies can then be tailored to each patient’s specific neurocognitive–behavioral profile, difficulties, and needs, supporting a shift toward more personalized care models in bvFTD. Patients in whom apathy and reduced conscientiousness are prominent may benefit most from structured environmental support, external cueing, routine-based interventions, and caregiver-mediated behavioral activation aimed at sustaining engagement and initiation. In contrast, when executive and attentional inefficiencies are more prominent, interventions targeting cognitive flexibility, task sequencing, planning, organization, and goal management may be particularly relevant. In many cases, combined and integrative intervention approaches may also be required.

### Strengths and limitations

A major strength of this study is the external validation of a theoretically grounded integrative model in a large independent cohort, enhancing confidence in its generalizability. Furthermore, the integration of penalized LASSO regression and structural equation modeling provides complementary insights into both predictor selection and underlying mechanistic pathways.

However, several limitations should be acknowledged. Variable selection was partially guided by prior findings from the Greek study, which may have constrained the exploration of alternative model configurations. For instance, episodic memory measures were not directly included in the present model, as they were not significantly associated with functional status in early-stage bvFTD in the previous study; however, their potential contribution in other samples or disease stages cannot be excluded. Similarly, the neuroimaging analysis was limited to a single prefrontal region, the right SFG, selected based on its correspondence with the right BA 8 identified in the original study, which may have limited our ability to identify additional brain–behavior relationships, particularly with regard to behavioral and personality-related measures. Another limitation concerns the comparability of assessment measures across the two cohorts. As this study was based on retrospectively collected data from an independent cohort at a different center, the same assessment instruments were not available across all domains. Therefore, variables were selected based on conceptual and psychometric comparability, with priority given to available measures assessing the same underlying constructs as those used in the original study. Although identical instruments were available for some domains, closely matched measures or proxies were used for others. Similarly, with respect to neuroimaging measures, the atlas-defined right SFG ROI and the right BA 8 identified in the original study are not identical entities; therefore, their correspondence represents an anatomically informed regional approximation rather than a direct one-to-one neuroimaging mapping. These differences in measurement correspondence across neuroimaging and neuropsychological domains should be considered when interpreting the extent of replication across the two cohorts. Additionally, genetic, neuropathological, and broader network-level factors were not included. Finally, the cross-sectional design precludes inference regarding causality of effects. Future prospective research should therefore incorporate longitudinal designs, larger samples, multimodal imaging, and biological markers to further refine and extend this framework.

## Conclusions

In conclusion, two partially distinct yet complementary processes appear to contribute to functional impairment in bvFTD: one reflecting the impact of frontal neurodegeneration on higher-order cognitive functions and global cognition that are essential for independent everyday functioning, and another involving motivational and personality-related changes that directly undermine engagement in everyday activities.

Together, these findings provide strong evidence for a multidomain, integrative model of functional impairment in bvFTD, indicating that disability cannot be fully explained by cognitive decline alone but instead emerges from the dynamic interplay of neural, cognitive, behavioral, and personality-related processes. This multidimensional perspective supports comprehensive approaches to the clinical assessment and management of bvFTD, with the ultimate goal of optimizing clinical care and improving quality of life for affected individuals.

## Data Availability

The datasets generated and/or analyzed during the current study are not publicly available due to privacy and ethical restrictions but are available from the corresponding author to any qualified investigator upon reasonable request, for the purposes of replicating the procedures and results.
